# The role of the orthopaedic surgeon in the COVID-19 era: cautions and perspectives

**DOI:** 10.1186/s40634-020-00255-5

**Published:** 2020-05-27

**Authors:** Luca Ambrosio, Gianluca Vadalà, Fabrizio Russo, Rocco Papalia, Vincenzo Denaro

**Affiliations:** Department of Orthopaedic and Trauma Surgery, Campus Bio-Medico University of Rome, Via Alvaro del Portillo 200, 00128 Rome, Italy

**Keywords:** Orthopaedic surgery, COVID-19, SARS-CoV-2, Coronavirus, Surgical indication, PPE, Education, Telemedicine, Surgical algorithm, Elective surgery

## Abstract

The current coronavirus disease 2019 (COVID-19) pandemic has revolutionized global healthcare in an unprecedented way and with unimaginable repercussions. Resource reallocation, socioeconomic confinement and reorganization of production activities are current challenges being faced both at the national and international levels, in a frame of uncertainty and fear. Hospitals have been restructured to provide the best care to COVID-19 patients while adopting preventive strategies not to spread the infection among healthcare providers and patients affected by other diseases. As a consequence, the concept of urgency and indications for elective treatments have been profoundly reshaped. In addition, several providers have been recruited in COVID-19 departments despite their original occupation, resulting in a profound rearrangement of both inpatient and outpatient care. Orthopaedic daily practice has been significantly affected by the pandemic. Surgical indications have been reformulated, with elective cases being promptly postponed and urgent interventions requiring exceptional attention, especially in suspected or COVID-19^+^ patients. This has made a strong impact on inpatient management, with the need of a dedicated staff, patient isolation and restrictive visiting hour policies. On the other hand, outpatient visits have been limited to reduce contacts between patients and the hospital personnel, with considerable consequences on post-operative quality of care and the human side of medical practice.

In this review, we aim to analyze the effect of the COVID-19 pandemic on the orthopaedic practice. Particular attention will be dedicated to opportune surgical indication, perioperative care and safe management of both inpatients and outpatients, also considering repercussions of the pandemic on resident education and ethical implications.

## Introduction

In December 2019, severe acute respiratory syndrome coronavirus 2 (SARS-CoV-2) broke out in Wuhan, China, causing clusters of severe respiratory illness and rapidly spreading across the country [26]. In a matter of weeks, several outbreaks were recognized in Italy, Spain, France and the USA until on March 11, 2020 the World Health Organization (WHO) declared the coronavirus disease 2019 (COVID-19) a global pandemic, with > 100,000 cases and 100 countries infected [53]. At the time of this writing, patients affected by COVID-19 exceeded 4 million globally, with approximately 280,000 deaths [52], becoming an unprecedented worldwide health issue. The need to control the spread of COVID-19 has forced national and international governments to implement socioeconomic measures including confinement, arrest of non-essential production activities and financial resources reallocation. Healthcare services have been reorganized to handle the COVID-19 crisis while continuing to safely guarantee urgent care to the general population.

Orthopaedic daily practice has been profoundly revolutionized by the pandemic. Most elective surgeries, accounting for a substantial part of orthopaedic activity, have been deferred ensuring that personal protective equipment (PPE), intensive care unit (ICU) beds and additional workforce would be redistributed to tackle the COVID-19 emergency. On the other hand, conditions including severe trauma, musculoskeletal tumors and infections, still necessitate urgent care and cannot be delayed. As surgery requires working in a confined space in close contact with the patient, the risk of infection transmission during the procedure and generally in the context of patient care, is reasonably high [7]. Therefore, orthopaedic routine must be reshaped in light of an appropriate surgical indication and COVID-19 index of suspicion, with considerable effects of inpatient management and outpatient visit rescheduling.

In this review, we aim to analyze the complex and continuously evolving effect of the COVID-19 pandemic on orthopaedic surgery. Surgical indications will be discussed based on patients’ underlying condition, comorbidities and COVID-19 index of suspicion. Particular attention will be dedicated to PPE protocols and nasopharyngeal swab indications to be adopted before, during and after surgery. Inpatient care, physical therapy, containment and early discharge strategies will be also addressed. Outpatient follow-up will be examined in light of a risk-benefit ratio, also considering the novel opportunities offered by telemedicine. Furthermore, repercussions on resident education and ethical perspectives will be debated.

### Perioperative management of the orthopaedic patient in the COVID-19 era

#### SARS-CoV-2 transmission and relevant protective measures

The major routes of SARS-CoV-2 transmission are through respiratory droplets and contact with contaminated surfaces [6]. In addition, exhalation of respiratory secretions during aerosol-generating procedures (AGPs: tracheal intubation, non-invasive ventilation, tracheotomy, cardiopulmonary resuscitation, manual ventilation before intubation and bronchoscopy) may produce highly virulent airborne particles [55]. Although symptomatic patients are the primary source of infection, asymptomatic subjects may also spread the disease and should not be neglected [8]. Therefore, maintaining an interpersonal distance ≥1 m is essential to minimize viral particle dissemination during social and clinical encounters [57]. SARS-CoV-2 may persist up to 3 h in aerosols, 24 h on cardboard and 2–3 days on plastic [50]. Thence, aeration of closed environments, appropriate use of PPE, frequent hand hygiene and surface decontamination are mandatory. According to the WHO, standard precautions should be universally applied and all patients should wear a medical mask in public areas [55].

To date, several types of face masks are available and are distinguished by different filter efficiencies. Surgical masks are designed to prevent intraoperative contamination and have not proven to protect from droplet spread in laboratory conditions [50]. However, the use of surgical masks has demonstrated to reduce the risk of influenza [38] and SARS-CoV [60] transmission, probably by arresting the diffusion of larger droplets. In a report from Ng, 85% of the providers in close contact with a COVID-19 patient was wearing a surgical mask and none was infected [34]. Despite the low evidence, a recent metanalysis attested that surgical masks and N95 respirators may provide a similar protection against viral respiratory infections during non-AGPs [9]. Therefore, the use of surgical masks during low-risk patient interactions may be encouraged in case of respirator shortages.

Respirators are designed to protect against droplets and aerosols and are classified upon the percentage of filtered particles ≥300 nm. In Europe, respirators are distinguished in filtering facepiece-1 (FFP1), FFP2 and FFP3 when filtering capacity is ≥80%, ≥94% and ≥ 99%, respectively. Similarly, the Centers for Disease Control and Prevention (CDC) defines filter efficiency indicating the percentage of filtered particles in the device nomenclature (i.e. a N95 mask filters 95% of ≥300 nm particles) [50]. Due to the higher protective potential, the WHO recommends that all healthcare workers should wear a respirator (≥FFP2/N95) when performing AGPs. In all other situations, wearing a surgical mask is reasonably safe when providing direct care to COVID-19 patients, especially in case of respirator scarcity [51, 58].

#### Surgical indication

According to the guidelines proposed by local institutions and international societies, including the American Academy of Orthopaedic Surgeons (AAOS) [1, 23] and the American College of Surgeons (ACS) [5], elective surgeries should be judiciously postponed depending on the local prevalence of COVID-19 and resource availability (PPE, ICU beds, respirators and personnel). Conducting “business as usual” is firmly discouraged as it may result in hazardous shortages of PPE and healthcare workforce in case of unexpectedly evolving conditions.

By definition, a procedure is considered elective when no short-term or long-term negative impact may be expected if surgical treatment is delayed. However, such denotation is subjective in nature, as reported pain and disability may significantly vary among orthopaedic patients, thus influencing the decisional process. Therefore, determining which procedures are strictly elective and which ones should be performed remains challenging. The Centers for Medicare & Medicaid Services (CMS) have proposed a 3-tiered system considering both the acuity of the surgical procedure and the underlying patient condition [11]. Tiers 1, 2 and 3 define low, intermediate and high acuity treatments which, if not provided, may result in a null, partial or significant increase in patient morbidity or mortality, respectively. Patients are further designated as “a” if healthy or “b” when unhealthy. The CMS recommends postponing Tier 1a operations (i.e. carpal tunnel release), considering deferral of Tier 2a procedures (i.e. joint replacement and spine surgery) and continuing to operate Tier 3a conditions (i.e. cancers, severe trauma and “highly symptomatic patients”). However, symptom severity is subjective and may generate unwanted ambiguity when formulating a surgical indication. To prevent any equivocacy, the Ohio Hospital Association imposed to cancel operations that did not match with the following criteria: “threat to the patient’s life if surgery or procedure is not performed, threat of permanent dysfunction of an extremity or organ system, risk of metastasis or progression of staging, risk of rapidly worsening to severe symptoms” [40]. Such principles may be useful when planning the restriction of surgical indications in case of paucity of resources during the peak of the pandemic.

Apart from treating trauma and tumors, Chang Liang et al. also allowed to operate on day surgical cases, including arthroscopies, implant removals and soft-tissue procedures. This early discharge policy may effectively reduce patients’ risk of nosocomial COVID-19 infection while not excessively weighing on healthcare resources. Conversely, elective procedures requiring > 23 h of hospitalization have been postponed and temporarily tackled with pain-relieving strategies [10].

According to different surgical indications and socioeconomic measures adopted during the pandemic, an overall diversification of orthopaedic cases compared to normal surgical routine should be expected. Quarantine, remote working and restriction of recreative activities will likely result in a reduction of vehicle accidents and work-related trauma, while school closure may increase the rate of pediatric injuries [29]. On the other hand, as elderly people will be more likely at home without the aid of caregivers, an increment of fractures due to domestic falls should be foreseen as well. Fractures in the elderly population, especially at the lower limbs, are associated with increased susceptibility to pulmonary infections and a considerable risk of mortality. In a retrospective study of 10 patients affected by COVID-19 and hospitalized for bone fractures, Mi et al. reported increased clinical severity and mortality after open reduction and internal fixation surgery. Hence, authors conclude that nonoperative treatment for fractures in the elderly should be considered in the first place when appropriate [33].

**Fig. 1:**
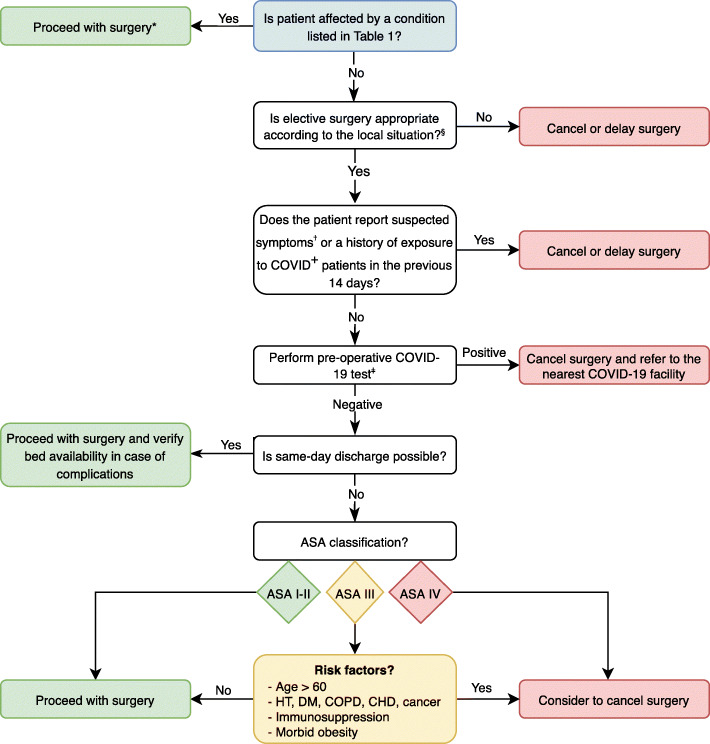
Decisional algorithm for guiding surgical indication during the COVID-19 pandemic. *A preoperative nasopharyngeal swab should be obtained and tested for SARS-CoV-2 as soon as reasonably possible without delaying surgery. ^§^Elective surgery indication should be judiciously pondered depending on availability of ICU beds, ventilators, PPE, workforce, blood units as well as institutional priorities. ^†^Suspected symptoms include fever and at least one sign/symptom of respiratory disease, e.g.*,* cough, shortness of breath [54].^ǂ^In case of a pending or undetermined result, consider whether to postpone surgery or to proceed adopting all the precautions for suspected or COVID^+^ patients. ASA: American Society of Anesthesiologists; HT: hypertension; DM: diabetes mellitus; COPD: chronic obstructive pulmonary disease; CHD: cardiac ischemic disease

Based on the guidelines provided by the AAOS [23], ACS [4, 5] and CMS [11], together with additional expert opinions [16, 19, 36, 42, 44, 62], we herein propose a decisional algorithm to assist the formulation of surgical indications in orthopaedic patients during the pandemic (Fig. 1). Conditions needing urgent care are listed in Table 1. It is advisable that the ultimate decision whether proceed or not to surgery is made by a multidisciplinary committee composed of surgery, nursing, anesthesia and administration representatives cautiously considering local COVID-19 prevalence, PPE supply, availability of workforce, ventilators and beds (including ICU) as well as patient age and comorbidities [37].

**Table 1:** Orthopaedic conditions needing urgent care [4, 11, 15, 16, 19, 23, 31, 36, 42, 46, 62]

Compartment syndrome	
Open fractures	
Periprosthetic fractures	
Joint dislocations	
Acute limb ischemia (including traumatic amputations)	
Septic arthritis	
Cauda equina Syndrome	
Hip and intertrochanteric fractures in the elderly	
Pelvic fractures	
Spinal cord compression or radiculopathy due to unstable vertebral fractures, spinal tumor, disc herniation, epidural abscess or hematoma with worsening neurological symptoms and/or intractable pain	
Complex fractures or long bone fractures that may lead to loss of function or permanent disability if treated non-operatively	
Necrotizing fasciitis	

#### Preoperative workup

Immediately after patient admission, COVID-19 risk profile and history of exposure should be thoroughly assessed [1]. In order to minimize the chance of nosocomial infections, same-day admission should be encouraged. Patients should be contacted the day before surgery and investigated for COVID-19 risk factors, including flu-like symptoms, travel history and harmful exposures in the previous 14 days [10]. Upon arrival, temperature should be checked and a surgical mask provided to all patients [37]. In accordance with local resources, all patients undergoing elective surgery should be preoperatively tested for COVID-19 [55]. In emergent cases where surgical treatment cannot be delayed, the test should be readily performed and processed as soon as reasonably possible [31].

COVID-19 testing requires an upper respiratory specimen obtained with a nasopharyngeal swab. The standard reference analysis detects viral RNA using real-time polymerase chain reaction (RT-PCR), a highly sensitive test providing results in 2–6 h. In areas with no known SARS-CoV-2 circulation, at least two different genome targets should be assessed. In case of discordance, the patient should be resampled. Conversely, in areas with a high SARS-CoV-2 circulation, a negative result in presence of a high index of suspicion does not exclude the diagnosis and requires additional analysis [56]. While waiting for the results, contact and droplet precautions should be adopted in addition to standard measures [55].

#### Operative room setting and intraoperative precautions

Suspected or confirmed COVID-19 cases should be treated in a dedicated space, away from busy zones and deprived of non-essential materials [47]. Operative personnel should be reduced to the minimum and unnecessary traffic in and out the OR should be discouraged. Sales representatives should be present only if strictly necessary [45]. Surgery should be performed in negative-pressure ORs to avoid the dissemination of the virus outside the theatre. However, ORs are usually equipped with positive-pressure systems to reduce the risk of surgical contamination. Therefore, as conversion to negative pressure may require OR maintenance, this should be planned with reasonable notice. If negative pressure cannot be obtained, positive pressure should be turned off and a portable high-efficiency particulate air (HEPA) filtration system with frequent air changes should be used [39].

Rodrigues-Pinto et al. proposed a workflow to safely operate in COVID-19-dedicated ORs identifying 5 main areas [39]:

1. Entry dressing room. Here essential PPE is donned, including: a disposable scrub suit, surgical boots or shoes, waterproof shoe covers, a surgical hood, goggles or a face shield and a respirator (FFP2/N95 are recommended) [20]. Hand hygiene should be performed before proceeding.
2. Anteroom. Here patient positioning should be optimized before hand scrubbing. An additional surgical hood should be worn along with non-sterile surgical gloves and a non-sterile gown to adjust the patient on the surgical bed. When indicated, prone positioning is preferable to reduce viral transmission through respiratory droplets [62]. Before sterile handwashing, non-sterile hood, gloves and gown should be removed. Lead garment should be worn if appropriate. After hand scrubbing, sterile disposable gowns and gloves are donned. An additional pair of gloves is recommended. If surgical helmets are employed, the use of a respirator cannot be disregarded. Indeed, unlike powered-air purifying respirators, surgical helmets mainly serve as liquid barriers and are not provided with HEPA filters [14]. If airway management is required, all surgical personnel not needed should remain outside the OR [3].
3. OR. The most appropriate surgical approach to reduce operative time should be considered. Although electrosurgical and high-speed devices (e.g. saw, drill) utilized during orthopaedic surgery are known to generate aerosols [59], limited data is currently available regarding the risk of virus spread. Therefore, electrocautery use should be minimized and power set at the lowest possible. Suction devices should always be employed to reduce surgical smoke and aerosols generated during motorized procedures [61]. Using absorbable sutures is advisable to diminish the need of additional post-operative visits. For the same reason, the use of a splint rather than a plaster to immobilize a limb is preferred [45]. In addition, transparent film dressings are useful when planning remote wound evaluation [37].
4. Exit room. Before leaving the OR, the surgeon should remove sterile gown and gloves and perform an accurate hand hygiene. Once in the exit room, PPE is sequentially removed, starting from the lead garment followed by the surgical hood, goggles, shoe covers and the respirator. Hand disinfection should be repeated after removing each piece of PPE.
5. Exit dressing room. Surgical personnel can change and leave the operative complex.

#### Postoperative care and inpatient management

After surgery, suspected or COVID-19^+^ patients should be transferred to an isolation room with contact and droplet precautions, or to the ICU if needed. In case of a negative test, patients may be routinely treated with standard precautions [55]. Several strategies to reduce contacts with inpatients have been proposed. Utilizing long-lasting wound dressings may reduce the need for repeated visits. Massey et al. proposed to position monitors and machines for intravenous drug administration outside patient rooms, so as to manage vital parameters, fluids and medications without the need to touch the patients [31]. Visiting hours should be restricted and a maximum of one visitor per room should be allowed. An early discharge strategy should be adopted whenever appropriate [37].

Departmental activity may be compartmentalized by establishing different teams [10]: (1) an inpatient team, responsible for visits in the ward, interdepartmental consultations and on-call services; (2) an outpatient team, deputed to attend urgent and undeferrable post-operative visits in the clinic; (3) a surgical team, devoted to operating on the cases that have been selected according to the criteria discussed above. This may be further divided into sub-teams consisting of different subspecialists (i.e. spine, knee, hip, shoulder, trauma surgeons) working on an in-house or on-call basis. Each team should rotate every 1 or 2 weeks, followed by a preventive isolation of 14 days. Moreover, teams should have dedicated workstations and avoid contacts among each other, in order to reduce the risk of cross-contamination [31]. If one provider desires not to return home after caring for COVID-19^+^ or suspected cases, healthcare institutions should provide the possibility for alternative temporary housing [37].

Following orthopaedic surgery, early physical therapy is fundamental to recover joint mobility, function and flexibility as well as to avoid the complications of prolonged immobilization. However, as physical therapists work in close contact with patients, COVID-19 poses a great risk towards their health as well. International societies recommend suspending physical therapy treatments for all orthopaedic issues excepting trauma and post-operative immobilization. Telerehabilitation should be encouraged for all non-essential treatments. If hypomobility might negatively impact on patient’s health, hands-on treatment may be considered but with adequate PPE [2, 35].

#### Outpatient care and telemedicine

During the pandemic, face-to-face visits should be limited to urgent cases and post-operative care that cannot be self-provided or remotely delivered. The latter include wound check, suture removal, evaluation of fracture reduction, highly symptomatic patients suspected for healing-related complications and follow-up visits that may likely change the management of the case [45]. All patients accessing the clinic should wear a face mask and undergo temperature check. In case of flu-like symptoms or exposure to confirmed or suspected cases, patients should be redirected to the emergency department for further evaluation [10]. Companions should not be allowed, except for non-ambulatory and disabled patients. All providers should perform frequent and accurate hand hygiene, adopt droplet precautions and wear appropriate PPE (a disposable gown, non-sterile gloves, a face shield or goggles, a FFP2/N95 respirator or a surgical mask if unavailable) [10].

In all cases not needing urgent face-to-face visits, telemedicine may be employed as a useful adjunct to minimize the spread of COVID-19 while ensuring continuous care [30]. In addition to phone consultations, telemedicine offers the possibility to perform remote virtual visits through the use of video-based platforms (e.g. Microsoft Teams™, Skype™). Such applications are now widely available and accessible by most smartphones and notebooks. This technology may be useful to triage new consults and conduct follow-up or non-urgent post-operative visits in quarantined patients [37]. Direct visualization may allow for a rapid inspection and implementation of wearable sensors may facilitate outcome assessment in certain situations (e.g. knee range of motion after total knee arthroplasty) [10]. In addition, these platforms can facilitate the diffusion of educational media, deliver outcome evaluation questionnaires and enhance patience rehabilitation [35]. Among these advantages, telemedicine also abates the use of PPE, reduces the risk of loss to follow-up and avoids that patients feel abandoned by their physician. However, the use of public virtual platforms raises concerns regarding privacy violations and unwanted data sharing. Thence, patients should be preventively informed about such risks before using third-party software [24]. Nevertheless, it is imperative to make patients aware that a virtual visit cannot replace face-to-face examination and the ultimate diagnosis of their condition.

### The impact of the COVID-19 pandemic on resident education

Due to the reduced volume of orthopaedic cases, several departments have adopted a “residency surge plan”, with a part of trainees committed to routine hospital duties and the remaining quarantined at home or redeployed in COVID-19-dedicated wards [28]. Disruption of orthopaedic residency routine, usually consisting of surgical training, inpatient and outpatient care, will likely have an enormous impact on resident education [13]. This is particularly relevant when considering that surgical training is practical in nature and is normally delivered in a climate of increasing autonomy, responsibility and complexity. Therefore, preserving orthopaedic education integrity while safeguarding resident health is a priority.

Schwartz et al. [43] have recently proposed a structured strategy to reorganize the orthopaedic residency program based on five basic principles:

1. Patient and provider safety: interpersonal distancing is required together with proper use of PPE and patient contact restricted to the minimum needed;
2. Provision of necessary care: orthopaedic residents must continue to participate in the diagnosis and treatment of musculoskeletal disorders;
3. System sustainability: resident workforce should be disposed to obtain the maximum output with minimum effort in respect of resource availability and institutional necessities;
4. System flexibility: the strategy should be tailored to the evolving pandemic and able to adapt to future unpredictable changes;
5. Preservation of command and control: hospital overload, redeployment in COVID-19 departments and disruption of the daily routine are posing a significant stress for residents and trainees. Emotional overwhelming, inadequacy and uncertainty of the future are all factors that may promptly lead to burnout and must be acquainted by program directors [18].

Bearing these principles in mind, residents may be divided in two teams: “active-duty” and “remotely working”. While “active-duty” members are mainly involved in clinical tasks, “remotely working” residents may support the active group with administrative assignments and bureaucratic practices. Whenever possible, clinical and surgical care should be limited to the faculty so as to reduce resident exposure, considering their front-line involvement in patient care [43].

Removal from routine orthopaedic duties inevitably interrupts the learning flow typical of residency. Therefore, program directors must provide residents with novel learning tools and possibilities. In this regard, virtual learning is an efficacious solution with multiple advantages, including the possibility to review recorded content, access imaging data and share relevant media without the need of personal contact. Apart from scheduled lectures, these platforms may be also employed to deliver case presentations, multidisciplinary meetings and conference talks. To date, several applications are available for this scope (e.g. Microsoft Teams™, Google Classroom™, Zoom™) [28].

The reduced surgical volume poses a double-edged condition to residents: whilst the absence of strict time constrains (as occurring during ordinary elective practice) may allow in-house trainees to acquire surgical techniques in a more relaxed environment, the overall decrease of surgical activity abates the chance of hands-on learning for most residents. In this regard, surgical simulation may be useful to improve practical skills away from the OR. Cadaveric dissection and procedural courses [25], virtual reality training [48] and arthroscopic simulators [21] are useful resources that may be improved and exploited to implement surgical education in the COVID-19 era. In addition, video-based education may further promote surgical training by providing audiovisual contents on indications, preoperative workup, OR setting, operative techniques and postoperative care [12]. Furthermore, diminished clinical and surgical demands offer the opportunity to intensify independent study, research activity and future career planning [28].

### Ethical considerations

Since millennia, the patient-physician relationship has been founded on mutual respect, empathy and shared decision-making, with the absolute priority of defending patients’ health. For orthopaedic surgeons, this implies selecting the most appropriate strategy to deal with pain and disability while considering patients’ comorbidities and expectations. Most often, this does not lead to save one’s life but to preserve his quality of life, which is not always less important. In presence of a global pandemic, each clinician is required to move his own focus from the individual to the collectivity, in order to satisfy the needs of the general population rather than the single patient. In this public health framework, a physician may not be allowed to do what he considers the best for his patients. In certain situations, some providers may be compelled to judge which patients should be intensively treated or not, thus incontrovertibly impacting on their prognosis [17].

As orthopaedic surgeons, appropriately selecting which patients should undergo surgery is essential not to drain vital resources from ICUs and COVID-19 departments. In case of clinical equipoise, i.e. when both conservative and operative management may likely lead to equivalent results, the former should be preferred whenever possible [37].

Preserving provider safety is essential to guarantee further care to the general population. To date, more than 500 healthcare providers have died of COVID-19 [32]. Fighting on the front line against SARS-CoV-2, especially when the limited availability of PPE cannot ensure an adequate protection, poses a vital risk on clinicians, especially the elder and the ones affected by serious comorbidities [22, 27]. This risk may be further increased in case of redeployment, which inevitably causes to work outside of one’s comfort zone, where errors are more likely to occur and lack of competency may lead to undecidedness, with terrible consequences [17, 41].

The COVID-19 pandemic imposes a significant psychological burden on every healthcare provider involved in the crisis [18]. In order to prevent such discomfort, it is imperative to develop proper protocols to rationalize personnel working hours and departmentalization, guide resource allocation and promote the belief that everyone is struggling for the greater good.

### What to expect from the future?

The COVID-19 pandemic has precipitated an unequalled global health crisis. Despite the prompt response in most countries, the different chronology of local outbreaks and the disparity among containment measures adopted pose a great hurdle to provide universal indications applicable for all facilities [49]. Furthermore, pandemic dynamics are continuously evolving thus needing careful monitoring and formulation of flexible dispositions that may be more or less permissive depending on COVID-19 prevalence, workforce availability and PPE supplies. Surgical indication should be continuously reassessed based on local, regional and national situations in accordance with both facility requirements and regulations from the authorities. Redeployment in COVID-19 units may be necessary and should not be disregarded, especially if reallocation might protect elder and weaker coworkers. Safety of all providers must be guaranteed with no exceptions. Nowadays, telemedicine offers incomparable advantages to remotely check our patients, although its limitations should not be neglected. Departmental activities should be adapted to actual clinical needs and education for resident and fellows should be promptly reorganized: they are the future of our profession.

COVID-19 has rapidly disrupted our routine in ways that would have been considered unconceivable in the contemporary era. Nonetheless, the fight against such a common enemy is awakening a sense of fraternity that is bringing the scientific community together with efforts never seen before. Whether these strategies are to be successful, history will judge us.

## Abbreviations

AAOS: American Academy of Orthopaedic Surgeons
ACS: American College of Surgeons
AGP: Aerosol-generating procedure
CDC: Centers for Disease Control and Prevention
CMS: Centers for Medicare & Medicaid Services
COVID-19: Coronavirus disease 2019
FFP: Filtering face piece
HEPA: High-efficiency particulate air
ICU: Intensive care unit
OR: Operating room
PPE: Personal protective equipment
RT-PCR: Real-time polymerase chain reaction
SARS-CoV-2: Severe acute respiratory syndrome coronavirus 2
WHO: World Health Organization

## References

[1] AAOS (2020). AAOS Clinical Considerations During COVID-19.

[2] AAPM&R (2020). COVID-19 statement from the American Academy of Physical Medicine & Rehabilitation Board of Governors.

[3] ACS (2020). COVID 19: Consideration for Optimum Surgeon Protection.

[4] ACS (2020). COVID-19 Guidelines for Triage of Orthopaedic Patients.

[5] ACS (2020). COVID-19: Recommendations for Management of Elective Surgical Procedures.

[6] Anfinrud Philip, Stadnytskyi Valentyn, Bax Christina E., Bax Adriaan (2020). Visualizing Speech-Generated Oral Fluid Droplets with Laser Light Scattering. New England Journal of Medicine.

[7] Ashford Robert U., Nichols Jennifer S., Mangwani Jitendra (2020). Annotation: The COVID-19 pandemic and clinical orthopaedic and trauma surgery. Journal of Clinical Orthopaedics and Trauma.

[8] Bai Yan, Yao Lingsheng, Wei Tao, Tian Fei, Jin Dong-Yan, Chen Lijuan, Wang Meiyun (2020). Presumed Asymptomatic Carrier Transmission of COVID-19. JAMA.

[9] Bartoszko JJ, Farooqi MAM, Alhazzani W, Loeb M (2020) Medical masks vs N95 respirators for preventing COVID-19 in health care workers a systematic review and meta-analysis of randomized trials. Influenza Other Respir Viruses. 10.1111/irv.12745 10.1111/irv.12745 PMC7298295 32246890

[10] Chang Liang Zhen, Wang Wilson, Murphy Diarmuid, Po Hui James Hoi (2020). Novel Coronavirus and Orthopaedic Surgery. The Journal of Bone and Joint Surgery.

[11] CMS (2020). Non-Emergent, Elective Medical Services, and Treatment Recommendations.

[12] Coe TM, Jogerts KM, Sell NM, Cassidy DJ, Eurboonyanun C, Gee D et al (2020) Practical techniques to adapt surgical resident education to the COVID-19 era. Ann Surg 10.1097/SLA.0000000000003993 PMC7224618 32675517

[13] Daodu OP, M.; Lopushinsky, S.; Varghese, T. K.; Brindle, M. (2020) COVID-19 – considerations and implications for surgical learners. Ann Surg 10.1097/SLA.0000000000003927 32345789

[14] Derrick JL, Gomersall CD (2004). Surgical helmets and SARS infection. Emerg Infect Dis.

[15] Di Martino A, Papapietro N, Lanotte A, Russo F, Vadala G, Denaro V (2012). Spondylodiscitis: standards of current treatment. Curr Med Res Opin.

[16] Donnally Chester J., Shenoy Kartik, Vaccaro Alexander R., Schroeder Gregory D., Kepler Christopher K. (2020). Triaging Spine Surgery in the COVID-19 Era. Clinical Spine Surgery.

[17] Dunham Alexandra M., Rieder Travis N., Humbyrd Casey J. (2020). A Bioethical Perspective for Navigating Moral Dilemmas Amidst the COVID-19 Pandemic. Journal of the American Academy of Orthopaedic Surgeons.

[18] Dyer GSM, Harris MB (2020) What's important: facing fear in the time of COVID-19. J Bone Joint Surg Am. 10.2106/JBJS.20.00469 10.2106/JBJS.20.00469 PMC7197343 32271211

[19] Ficke JR (2020). Orthopaedic Surgery Case Triage.

[20] Forrester Joseph D., Nassar Aussama K., Maggio Paul M., Hawn Mary T. (2020). Precautions for Operating Room Team Members During the COVID-19 Pandemic. Journal of the American College of Surgeons.

[21] Gomoll AH, O'Toole RV, Czarnecki J, Warner JJ (2007). Surgical experience correlates with performance on a virtual reality simulator for shoulder arthroscopy. Am J Sports Med.

[22] Guo Xiaodong, Wang Jiedong, Hu Dong, Wu Lisha, Gu Li, Wang Yang, Zhao Jingjing, Zeng Lian, Zhang Jianduan, Wu Yongchao (2020). Survey of COVID-19 Disease Among Orthopaedic Surgeons in Wuhan, People’s Republic of China. The Journal of Bone and Joint Surgery.

[23] Guy DK, Bosco JA, Savoie FH (2020). AAOS Guidelines for Elective Surgery During the COVID-19 Pandemic.

[24] Halawi Mohamad J., Wang Daniel D., Hunt Thomas R. (2020). What’s Important. The Journal of Bone and Joint Surgery.

[25] Holland JP, Waugh L, Horgan A, Paleri V, Deehan DJ (2011). Cadaveric hands-on training for surgical specialties: is this back to the future for surgical skills development?. J Surg Educ.

[26] Huang C, Wang Y, Li X, Ren L, Zhao J, Hu Y (2020). Clinical features of patients infected with 2019 novel coronavirus in Wuhan, China. Lancet.

[27] Jella TK, Acuna AJ, Samuel LT, Jella TK, Mroz TE, Kamath AF (2020) Geospatial Mapping of Orthopaedic Surgeons Age 60 and Over and Confirmed Cases of COVID-19. J Bone Joint Surg. 10.2106/JBJS.20.00577 10.2106/JBJS.20.00577 PMC7224628 32332218

[28] Kogan Monica, Klein Sandra E., Hannon Charles P., Nolte Michael T. (2020). Orthopaedic Education During the COVID-19 Pandemic. Journal of the American Academy of Orthopaedic Surgeons.

[29] Lockey SD (2020) What's important: what is our role in the COVID-19 pandemic? J Bone Joint Surg Am. 10.2106/JBJS.20.00444 10.2106/JBJS.20.00444 PMC7197342 32282413

[30] Loeb Alexander E., Rao Sandesh S., Ficke James R., Morris Carol D., Riley Lee H., Levin Adam S. (2020). Departmental Experience and Lessons Learned With Accelerated Introduction of Telemedicine During the COVID-19 Crisis. Journal of the American Academy of Orthopaedic Surgeons.

[31] Massey Patrick A., McClary Kaylan, Zhang Andrew S, Savoie Felix H., Barton R. Shane (2020). Orthopaedic Surgical Selection and Inpatient Paradigms During the Coronavirus (COVID-19) Pandemic. Journal of the American Academy of Orthopaedic Surgeons.

[32] Medscape (2020). In Memoriam: Healthcare Workers Who Have Died of COVID-19.

[33] Mi Bobin, Chen Lang, Xiong Yuan, Xue Hang, Zhou Wu, Liu Guohui (2020). Characteristics and Early Prognosis of COVID-19 Infection in Fracture Patients. The Journal of Bone and Joint Surgery.

[34] Ng K, Poon BH, Kiat Puar TH, Shan Quah JL, Loh WJ, Wong YJ et al (2020) COVID-19 and the risk to health care workers: a case report. Ann Intern Med. 10.7326/L20-0175 10.7326/L20-0175 PMC7081171 32176257

[35] Pedersini P, Corbellini C, Villafane JH (2020) Italian physical Therapists’ response to the novel COVID-19 emergency. Phys Ther. 10.1093/ptj/pzaa060 10.1093/ptj/pzaa060 PMC7184495 32280973

[36] Placella G, Salvato D, Delmastro E, Bettinelli G, Salini V (2020) CoViD-19 and Ortho and trauma surgery: the Italian experience. Injury 10.1016/j.injury.2020.04.012 PMC7159876 32327236

[37] Prada C, Chang Y, Poolman R, Johal H, Bhandari M (2020). Best Practices for Surgeons. COVID-19 Evidence-Based Scoping Review. A Unifying Report of Global Recommendations.

[38] Radonovich LJ, Simberkoff MS, Bessesen MT, Brown AC, Cummings DAT, Gaydos CA (2019). N95 respirators vs medical masks for preventing influenza among health care personnel: a randomized clinical trial. JAMA.

[39] Rodrigues-Pinto R, Sousa R, Oliveira A (2020) Preparing to perform trauma and Orthopaedic surgery on patients with COVID-19. J Bone Joint Surg Am. 10.2106/JBJS.20.00454 10.2106/JBJS.20.00454 PMC7197341 32282412

[40] Sarac NJ, Sarac BA, Schoenbrunner AR, Janis JE, Harrison RK, Phieffer LS et al (2020) A review of state guidelines for Elective Orthopaedic procedures during the COVID-19 outbreak. J Bone Joint Surg Am. 10.2106/JBJS.20.00510 10.2106/JBJS.20.00510 PMC7197340 32282419

[41] Sarpong NO, Forrester LA, Levine WN (2020) What’s important: redeployment of the Orthopaedic surgeon during the COVID-19 pandemic: perspectives from the trenches. J Bone Joint Surg Am. 10.2106/JBJS.20.00574 10.2106/JBJS.20.00574 PMC7224625 32287087

[42] Schmidt T (2020). Elective Surgery Algorithm.

[43] Schwartz AM, Wilson JM, Boden SD, Moore TJJ, Bradbury TLJ, Fletcher ND (2020). Managing resident workforce and education during the COVID-19 pandemic: evolving strategies and lessons learned. JBJS Open Access.

[44] Stahel PF (2020). How to risk-stratify elective surgery during the COVID-19 pandemic?. Patient Saf Surg.

[45] Stinner DJ, Lebrun C, Hsu JR, Jahangir AA, Mir HR (2020) The Orthopaedic trauma service and COVID-19 - practice considerations to optimize outcomes and limit exposure. J Orthop Trauma. 10.1097/BOT.0000000000001782 10.1097/BOT.0000000000001782 PMC7188036 32301767

[46] Tang Pei-Fu, Hou Zhi-Yong, Wu Xin-Bao, Zhang Chang-Qing, Wang Jun-Wen, Xing Xin, Shao Zeng-Wu, Yu Ai-Xi, Wang Gang, Chen Bin, Zhang Ping, Hu Yan-Jun, Wang Bo-Wei, Guo Xiao-Dong, Tang Xin, Zhou Dong-Sheng, Liu Fan, Chen Ai-Mi, Zhang Kun, Li Kai-Nan, Zhu Yan-Bin (2020). Expert consensus on management principles of orthopedic emergency in the epidemic of coronavirus disease 2019. Chinese Medical Journal.

[47] Ti Lian Kah, Ang Lin Stella, Foong Theng Wai, Ng Bryan Su Wei (2020). What we do when a COVID-19 patient needs an operation: operating room preparation and guidance. Canadian Journal of Anesthesia/Journal canadien d'anesthésie.

[48] Vadala G, De Salvatore S, Ambrosio L, Russo F, Papalia R, Denaro V (2020). Robotic spine surgery and augmented reality systems: a state of the art. Neurospine.

[49] Vannabouathong Christopher, Devji Tahira, Ekhtiari Seper, Chang Yaping, Phillips Steven A., Zhu Meng, Chagla Zain, Main Cheryl, Bhandari Mohit (2020). Novel Coronavirus COVID-19. The Journal of Bone and Joint Surgery.

[50] Viswanath Aparna, Monga Puneet (2020). Working through the COVID-19 outbreak: Rapid review and recommendations for MSK and allied heath personnel. Journal of Clinical Orthopaedics and Trauma.

[51] WHO (2020). Advice on the use of masks in the context of COVID-19.

[52] WHO (2020). Coronavirus disease 2019 (COVID-19) situation report-112.

[53] WHO. Coronavirus disease 2019 (COVID-19) situation report–51. 2020; https://www.who.int/docs/default-source/coronaviruse/situation-reports/20200311-sitrep-51-covid-19.pdf?sfvrsn=1ba62e57_10

[54] WHO (2020). Global surveillance for COVID-19 caused by human infection with COVID-19 virus.

[55] WHO (2020). Infection prevention and control during healthcare when COVID-19 is suspected.

[56] WHO (2020). Laboratory testing for coronavirus disease 2019 (COVID-19) in suspected human cases: interim guidance.

[57] WHO (2020). Q&A on coronaviruses (COVID-19).

[58] WHO (2020). Rational use of personal protective equipment for coronavirus disease (COVID-19) and considerations during severe shortages.

[59] Yeh HC, Turner RS, Jones RK, Muggenburg BA, Lundgren DL, Smith JP (1995). Characterization of aerosols produced during surgical procedures in hospitals. Aerosol Sci Technol.

[60] Yen MY, Lin YE, Lee CH, Ho MS, Huang FY, Chang SC (2011). Taiwan’s traffic control bundle and the elimination of nosocomial severe acute respiratory syndrome among healthcare workers. J Hosp Infect.

[61] Zheng MH, Boni L, Fingerhut A (2020) Minimally invasive surgery and the novel coronavirus outbreak: lessons learned in China and Italy. Ann Surg. 10.1097/SLA.0000000000003924 10.1097/SLA.0000000000003924 PMC7188059 32221118

[62] Zou J, Yu H, Song D, Niu J, Yang H (2020). Advice on standardized diagnosis and treatment for spinal diseases during the coronavirus disease 2019 pandemic. Asian Spine J.

